# Role of skin-homing t-cells in recurrent episodes of atopic dermatitis: a review

**DOI:** 10.3389/fimmu.2025.1489277

**Published:** 2025-02-18

**Authors:** Huimin Guo, Huimin Yuan, Yanru Yu, Jingwei Sun, Yan Sun, Yang Tang, Fengjie Zheng

**Affiliations:** ^1^ School of Traditional Chinese Medicine, Beijing University of Chinese Medicine, Beijing, China; ^2^ Beijing Tsinghua Changgung Hospital, School of Clinical Medicine, Tsinghua University, Beijing, China

**Keywords:** atopic dermatitis, pathogenesis, T lymphocytes, skin-homing, recurrence

## Abstract

Atopic dermatitis (AD) is a chronic relapsing disease with complex pathogenesis. Among them, inflammation is one of the primary pathogenesis of AD. AD is characterized by infiltration of lymphocytes into the skin’s dermis, and the skin homing of lymphocytes plays an essential role in the recurrence of AD. Currently, there is more and more evidence to support this view. This article reviews the relevant role of T lymphocyte skin-homing-related molecules in the recurrence of AD to provide a reference for the cure of AD.

## 1 Introduction

Atopic dermatitis (AD), clinically characterized by recurrent episodes, dry skin, intractable itching, and chronic eczematoid lesions, is one of the most common chronic, inflammatory, and recurrent skin diseases (1, 2). The incidence of AD is high and increasing year by year (3). In 2019, the number of cases of AD worldwide was 171 million, an increase of 28.6% from 133 million cases in 1990 (4). At present, sudden recurrence or deterioration of symptoms (68% of AD patients) is one of the most challenging factors in the course of AD (5), and the main obstacle in AD research and treatment. The pathogenesis of AD is complex, involving the combined disruption and imbalance of many factors, such as microbial distribution, genetics, skin barrier, immune response, and so on (6–9). However, there is still controversy regarding how AD begins. There are mainly two hypotheses: “outside-in” (immune imbalance caused by epidermal skin barrier destruction) and “inside-out” (systemic inflammation triggers barrier dysfunction) (10). Studies have shown that the intensity of barrier damage and water deficit of non-pathological AD skin is closely related to the clinical severity of AD patients. This strongly suggests that the destruction of the steady state of the percutaneous permeability barrier is caused by induced dermatitis, which then triggers the tendency to induce AD recurrence (11). Patients with AD frequently relapse in areas where the primary lesions have receded after cessation of treatment and studies have also shown that there is a correlation between the mechanism of recurrence and local immune memory function (12). Therefore, the “inside-out” hypothesis may provide crucial insights into the mechanisms of AD recurrence.

AD is characterized by infiltration of multiple immune cells, primarily lymphocytes, into the dermis. After antigen exposure, naïve T cells differentiate into effector T cells capable of executing immune defense mechanisms. Most of these cells are transient and die after an immune response, but some still exist and differentiate into memory T cells. When AD relapses, the Effector Memory T Cells (TEM) circulating in the blood migrate rapidly to the skin (13). In this process, the lymphocytes circulating in the blood selectively cross the high endothelial venules, migrate directionally and enter peripheral organs or specific tissue regions. This phenomenon is called lymphocyte homing (14, 15). Current research on skin-homing memory T cells is mainly focused on TEM.

**Figure 1:**
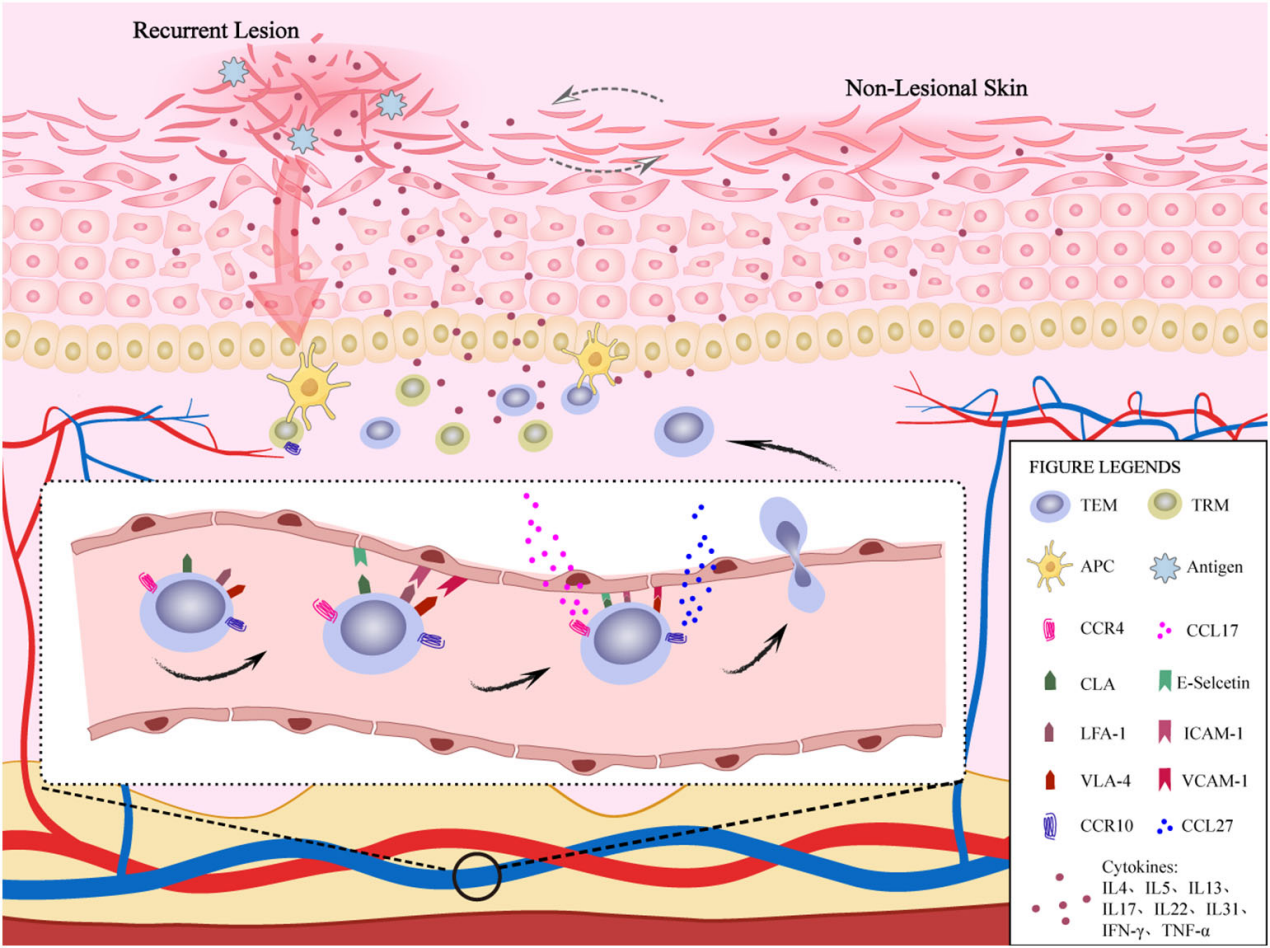
The role of TEM homing to the skin in AD recurrence. TEMs in blood vessels achieve adhesion and rolling of lymphocytes on high endothelial venules through the interaction of CLA, LFA-1, and VLA-4 molecules on their surface, as well as their ligands E-selectin family, intercellular adhesion molecule-1 and vascular cell adhesion molecule-1 on the surface of vascular endothelial cells. Subsequently, CCR4 and CCR10 expressed on TEMs interact with CCL17 and CCL27 produced by keratinocytes. TEMs eventually stay and cross endothelial cells to achieve skin homing and produce cytokines, which further disrupt the skin barrier, leading to the recurrence of AD. In addtion, TRMs also participate in the immune response and produce cytokines during AD recurrence. In non-lesional skin, there is also a slight inflammatory response.

TEM leaves the vascular system to enter the dermis for homing and must cross the dermal microvascular endothelial cell “barrier”. This is a multi-step process that requires additional molecular interactions to mediate. The first step is composed of cutaneous lymphocyte-associated antigen (CLA), lymphocyte-associated antigen-1 (LFA-1), very late appearing antigen-4 (VLA-4), etc, as well as their ligands on the surface of vascular endothelial cells, such as E-selectin, intercellular cell adhesion molecule-1 (ICAM-1), vascular cell adhesion molecule-1 (VCAM-1) and other interactions to achieve the adhesion and rolling of lymphocytes on high endothelial micro vessels (16). The second step is activated through the interaction of CCL17 (C-C chemokine ligand 17) and CCL27, with their receptors CCR4 and CCR10, where the lymphocytes ultimately lodge and cross the endothelium (17). The rapid homing of the CLA+ T cell immune response to the skin results in production of Th2 cytokines, further disrupting the skin barrier, leading to recurrence of AD (**Figure 1**). Clinical studies have shown that from the cessation of dupilumab treatment to 48 weeks after treatment, CLA+ T cells produced a significant increase in Th2-related cytokines, indicating that the recurrence process is closely related to CLA+ T cells (18). This article reviews the relevant role of TEM skin-homing-related molecules in the recurrence of AD, proving a reference for the cure of AD.

## 2 The adhesion molecules

Cell adhesion between TEM and vascular endothelial cells is an important early step in the recurrence of AD, which is mainly involved in CLA and integrin family.

### 2.1 CLA and E-selectin

CLA is a cell surface molecule induced by fucosyl transferase VII. It is a ligand of glycoprotein E-selectin that is expressed on the surface of most peripheral blood leukocytes. In the case of inflammation, CLA is expressed on 90% of TEM in the skin but not on naive T cells (19, 20). CLA+ T cells are T cells specific to skin homing, of which CD3+CD4+CD45RO+CLA+ T cells are the central infiltrating cells in AD lesions (21–23). When the lectin structural domain of E-selectin on endothelial cells recognizes CLA, CLA functions as an adhesion molecule (20), allowing T lymphocytes to enter the inflamed skin site by rolling across the vascular endothelium (24). The infiltration of huge amounts of CLA+ T cells in the skin of AD lesions (21) indicate that circulating CLA+ T cells can be used as a peripheral biomarker of AD (19).

The importance of circulating CLA+ T cells in dermatology depends on their ability to selectively migrate to the skin (homing) and their de-homing ability, suggesting that these CLA+ T cells may reflect the skin’s immune response (25). Efalizumab can improve the clinical symptoms of AD by blocking the interaction of homing adhesion molecules (26). During treatment, patients developed secondary CLA+ T cell increase, and the disease worsened after treatment is discontinued, indicating that T cell recycling/turnover between skin and blood is regular. In this case, TRM can migrate from the skin back to the blood (27), exhibit CLA+ Th2 characteristics and increased expression of GATA binding protein 3 and interleukin-13 (IL-13) (28). The de-homing characteristic of circulating CLA+ T cells increases the positive correlation between the phenotype and number of circulating CLA+ T cells and the severity of AD (29).

### 2.2 Integrin family

Integrins are a large family of widely expressed adhesion receptors interacting with cell surface homologous receptors and extracellular matrix components. Integrin affinity can be regulated by elements of the extracellular environment or by intracellular signals (30). During homing, lymphocytes stagnate on the vascular endothelium and migrate spontaneously in a rapid crawling mode to find the best extravasation locations (portals) (31). These portals are composed of endothelial cells with vertical microvilli-like protrusions rich in adhesion molecules, especially integrin ligands ICAM-1 and VCAM-1. LFA-1 and VLA-4 are members of integrin-type adhesion molecules involved in the adhesion and rolling process of lymphocyte extravasation during lymphocyte homing to the skin in AD (32).

#### 2.2.1 Integrin LFA-1 and its ligand ICAM-1

ICAM-1 is a member of the immunoglobulin superfamily and is the primary ligand of LFA-1. It is enriched in nearly 43% of the portals on the vascular endothelium. Sending a signal to the nearby portal prepares endothelial cell connections to adapt to TEM passage, making these portals more suitable for TEM migration to achieve homing (31). The study found that the vascular endothelium in the dermis showed a strong signal of ICAM-1 in AD patients compared with normal people (33), indicating that ICAM-1 plays a vital role in the TEM homing of AD. The regulation of the interaction between CLA+ T cells and vascular endothelial cells to promote their adhesion by the ICAM-1/LFA-1 adhesion system is one of the mechanisms of transendothelial migration of CLA+ T cells (34). The effect of ICAM-1/LFA-1 on lymphocyte skin homing is not only found in AD but also other inflammatory diseases involving the up-regulation of cell adhesion molecules. The expression of adhesion molecules ICAM-1, VCAM-1, LFA-1, and VLA-4, which promote leukocytes to enter the inflammatory site, were up-regulated in microvascular endothelial cells and leukocytes in the poor healing of skin ulcers caused by chronic venous insufficiency (35). In follicular keratosis, when epidermal keratinocytes do not express ICAM-1, the expression of ICAM-1 is upregulated in the follicular epithelium adjacent to LFA-1-positive follicular cutaneous T-cell lymphoma cells. This suggests that the ICAM-1/LFA-1 interaction plays a vital role in the pathogenesis of hair follicle keratosis (36).

The interaction between LFA-1 and ICAM-1 is necessary for lymphocyte adhesion and migration and plays a vital role in antigen presentation in AD (37). Clinical trials of AD have shown that LFA-1 blockade can inhibit the presentation of allergen-specific Th2 cells by keratinocytes at doses present *in vivo (* 38). Tsuyoshi Ohmura et al. (39) have also shown that preventive treatment can inhibit the development of AD-like lesions in mice, suggesting that anti-LFA-1 monoclonal antibodies may play a role by inhibiting antigen presentation.

The interaction between LFA-1 and ICAM-1 plays a vital role in the recurrence of AD. Many existing treatments can directly or indirectly down-regulate the level of ICAM-1 in AD. Anti-adhesion molecule therapy is becoming a new method for treating inflammatory skin diseases like AD.

#### 2.2.2 Integrin VLA-4 and VCAM-1

VLA-4 is a member of the VLA subfamily, composed of α4 and β1 subunits, and is highly expressed in almost all lymphocytes (40). The interaction between VLA-4 and cytokine-induced VCAM-1 not only mediates the initial tethering of cells but also mediates the firm adhesion of cells to the inflammatory vascular wall and plays a vital role in the migration of lymphocytes, monocytes, and eosinophils to the inflammatory site (32). *In vitro* AD experiments, monoclonal antibodies that block the interaction of CLA/E-selectin or VLA-4/VCAM-1 can significantly inhibit the transendothelial migration of skin-homing T cells (41). Similar findings were also found in rats, where when the two integrins VLA-4 and LFA-1 were blocked, lymphocyte accumulation was almost entirely inhibited (42). Although VCAM-1 is involved in the transendothelial migration of skin-homing T cells in AD, studies have shown no correlation between the soluble VCAM-1 concentration and the clinical severity of AD patients (43, 44). Therefore, whether they are indicators of disease activity still needs to be determined.

The interaction between VLA-4 and cytokine-induced VCAM-1 is not only involved in cell adhesion and migration but may also be involved in the adhesion and interaction between T lymphocytes and activated fibroblasts in the chronic inflammatory state of the skin (45). Studies have found that VCAM-1 is also expressed on skin keratinocytes and dendritic cells (46). In some cases, VCAM-1 on these cells is up-regulated by cytokines such as IL-4, tumor necrosis factor-α, IL-1, and interferon-γ, suggesting that VLA-4/VCAM-1 may play a role in the interaction between mononuclear leukocytes and connective tissue during inflammation, and this process is partially regulated by cytokines (45).

## 3 Chemokines

Chemokines are small proteins that play a significant role in controlling leukocyte transport. According to the cystine motifs with different numbers of amino acids between cysteine residues, they are divided into four subgroups: CC, CXC, CX3C, and C. Chemokine receptor is subdivided in the same way, such as CCR1 ± 9, CXCR1 ± 5, and CX3CR1 (47). The interaction between chemokines and their receptors in the blood plays a vital role in mediating T cells’ firm adhesion in the activation and transport process, mediating the infiltration of circulating T cells to the periphery of inflammation (48). In addition to the CLA and integrin family mentioned above, chemokines represented by CCR4 and CCR10 are also involved in mediating skin T cell transport in inflammatory skin diseases such as AD, psoriasis, and allergic contact dermatitis (49, 50).

### 3.1 CCL17 and its receptor CCR4

CCL17 is a member of the CC chemokine family, which is synthesized by various skin-derived cells such as keratinocytes, activated macrophages, dendritic cells and endothelial cells, and binds to CCR4 receptor (51, 52). CCR4 is highly expressed in skin infiltrating lymphocytes (52) and preferentially expressed on circulating CD4+ memory T cells and regulatory T cells (47). Studies have shown that *in vivo* CCR4+ memory CD4+ lymphocytes migrate more to dermal inflammation than CCR4- lymphocytes (53), and keratinocytes in the skin venules and epidermis constitutively and induciblely express CCL17 (54). Therefore, CCL17 and CCR4 are important in lymphocyte-selective skin homing (50, 51), and both are synergistically involved in the interaction between TEM homing to the skin and the site of skin inflammation and vascular endothelium (55).

Wang et al. (56) found that T cells with CCR4+ and CCR10+ increased in the skin and draining lymph nodes of allergic contact dermatitis and were effectively attracted by their specific chemokines CCL17, CCL22, and CCL27 *in vitro*. Using *in vivo* imaging technology, it was found that T cells migrated to the inflammatory site 2 hours after administration. At the same time, systemic administration of anti-CCR4 ligand (CCL17 and CCL22) and CCR10 ligand (CCL27) comprehensive antibody can significantly inhibit T cell migration and skin inflammation. In AD patients and mouse models, CCL17 is produced by basal keratinocytes (47, 57), and CCL17 mRNA is present in the endothelial cells of the skin’s postcapillary venules (55). In addition, many studies have found that serum CCL17 levels in AD patients increased when the disease worsened, and the number of CCR4+CLA+ circulating lymphocytes also increased (47, 53, 58, 59). These results suggest that CCL17 and its receptor may be vital in recruiting AD skin-specific lymphocytes.

CCL17 has been shown to be associated with the severity of AD disease and has been described as a biomarker that reflects AD treatment (60). Therefore, inhibiting the binding of CCL17 to CCR4 may prevent Th2 cells from migrating to inflammatory tissues and make CCR4 and CCL17 a potential target for the treatment of AD. Recent studies have shown that CCR4 deletion or CCR4 antagonist can improve AD-like skin lesions in BALB/c mouse AD model (61, 62). Recently, RPT193, an oral small molecule CCR4 antagonist, inhibited the migration of Th2 cells derived from healthy human CD4+ T cells in an *in vitro* chemotaxis assay. In moderate to severe AD subjects, RPT193 improved clinical efficacy more than placebo (63). Nelly Frossard et al. (64) found in the experiments that GPN279 can effectively improve the skin barrier and physiological indicators of patients with mild to moderate AD (GPN279, a chemical neutralizing agent, was recently found to bind CCL17 with high affinity and effectively neutralize CCL17, thereby activating the CCR4 receptor expressed by Th2 cells). The above studies have shown the effectiveness and clinical potential of CCR4 and CCL17 in the treatment of AD. In recent years, inhibiting the combination of CCR4 and CCL17 is emerging as a new method for treating AD.

### 3.2 CCL27 and its receptor CCR10

CCL27 is a skin-specific CC chemokine, constitutively expressed by keratinocytes and fixed on the surface of dermal extracellular matrix and dermal endothelial cells. Together with CCR4, it is involved in mediating lymphocytes to cross vascular endothelial cells, and is significantly expressed in the lesions of inflammatory skin diseases such as human AD (65), contact dermatitis, and psoriasis. CCR10 is a receptor for CCL27. In human subjects, all blood CCR10+ T cells showed memory cell markers, co-expressed the skin-homing molecule CLA, and responded to the chemotaxis of CCL27 (66). The CCR10/CCL27 interaction mediates the recruitment of memory T cells to the skin and regulates the induction of antigen-specific skin inflammation *in vivo (* 67). In early studies, it has been proven that keratinocytes of inflamed skin lesions of AD patients is positive for CCL27, with an increased level of serum CCL27, this increase is positively correlated to the severity of the disease (68). The chemotaxis and migration experiments of the AD mouse model showed that CCL27 promoted a greater degree of skin homing of T cells in diseased mice. Subcutaneous injection of neutralizing anti-CCL27 antibody to AD mice with early skin lesions can alleviate the clinical progression of inflammation with reduced infiltration of T cells and mast cells in the skin and down-regulation of inflammatory cytokines (69). The above studies suggest that the interaction between CCL27 and CCR10 is essential in promoting lymphocyte skin homing and hence AD recurrence. To further clarify the role of CCL27 and CCR10 in skin inflammation, Shinji Kagami et al. (70) found that although CCL27 alone is not enough to induce inflammation in the CCL27 transgenic AD mouse model, if inflammation shows a more robust Th2 shift response, the interaction between CCL27 and CCR10 will enhance skin inflammation, which may be produced by attracting Th2 cells expressing CCR4 into the skin. Therefore, CCL27 may be involved in the pathogenesis of skin diseases such as AD by regulating chronic allergic inflammation. In addition, CCL27 and CCR10 may be targets for developing new and selective treatments of inflammatory autoimmune skin diseases represented by AD.

Although the above CCL17/CCL27 and its receptor CCR4/CCR10 have been shown to be associated with the onset of a variety of skin allergies and inflammatory diseases, their regulatory effects on skin T cells *in vivo* are still unclear and sometimes controversial. *In vivo*, homing assay, functional blockade of CCL27 by anti-CCL27 monoclonal antibody prevented CCR4-deficient T cells from migrating to the site of skin inflammation but not in wild-type mice (58). Similarly, after treatment with CCR4 antagonists in AD model dogs, the clinical signs of some dogs (5/13) were partially inhibited (71). These studies suggest that there may be functional redundancy between CCR4 and CCR10. Interestingly, another study using anti-CCL27 monoclonal antibody alone was sufficient to prevent skin-specific T cell homing in wild-type AD mice (67), suggesting that CCR4 has no apparent redundancy and T cell transport to the skin only requires CCR10 to be achieved. However, the expression of CCR4 and CCR10 in CD4+ T cells from wild-type mice was directly compared under the same conditions. Some found that CCR4 deletion reduced the accumulation of memory CD4+ T cells in the skin by about 20 times, but CCR10 deletion did not produce any detectable effect. This study shows that the role of CCR10 in skin T cell immunity is unclear (72). Therefore, the regulatory impact of CCR4 and CCR10 on lymphocyte skin homing *in vivo* remains to be further studied. In any case, whether there is functional redundancy between CCR4 and CCR10 or not, blocking multiple pathways at the same time should be beneficial in the treatment of T cell-mediated skin diseases (49, 54).

## 4 Other related molecules and pathways

In addition to the above factors, cytokines are also involved in the homing of TEM in AD. Cytokines are the primary regulators of CLA expression and cytokine synthesis phenotype during memory T cell differentiation. To date, 32 ILs have been defined, some of which are involved in vascular regulation, play an important role in lymphocyte homing to the skin, and play a synergistic role in AD. IL-1 promotes the activation of dermal microvascular endothelial cells by up-regulating ICAM-1 or E-selectin (45). IL-4 can induce CCL17 and CCL22. IL-6 is produced by endothelial cells and acts on endothelial cells by activating IL-6 receptors. IL-6, as a general early pro-inflammatory mediator, is not limited to AD. IL-12 activates the influential up-regulation factor of CLA expression on T cells (73). IL-13 can induce CCL17 and CCL22, and the expression of IL-13 in skin-homing cells can be used as a marker of AD severity (23). IL-17 can induce the secretion of IL-1 and the up-regulation of CAMs such as ICAM-1 in dermal endothelial cells. The increase of TNF-α contributes to the up-regulation of chemokine expression, as well as the expression of adhesion molecules (E-Selectin, VCAM-1, and ICAM-1) produced by endothelial cells (45, 74). These cytokines are related to the skin homing of TEM and are closely related to the recurrence of AD.

Furthermore, available evidence suggests that the OX40-OX40L axis plays a crucial role in the pathogenesis of AD (75). OX40 is a co-stimulatory immune checkpoint molecule that promotes T cell differentiation and proliferation and the survival of multiple subsets of helper T cells by interacting with ligands (OX40L) on antigen-presenting cells (76). In addition, the OX40L/OX40 signaling axis can also increase the activity of OX40L cell types and increase the production of cytokines (77). More interestingly, studies have shown that increased OX40 expression is observed on skin homing T cells in AD patients (75), suggesting that the OX40-OX40L axis may play an important role in both TEM skin homing and in the recurrence of AD.

## 5 JAK/STAT pathway

The Janus kinase (JAK) signal transduction and activators of transcription (STAT) pathway (JAK/STAT pathway) is one of the essential pathways in inflammatory diseases such as AD (78). The JAK series includes JAK1, JAK2, JAK3 and TYK2, and the STAT series includes STAT1, STAT2, STAT3, STAT5A/B and STAT6. AD is known to be a biphasic T cell-mediated inflammatory disease, in which the acute phase is mainly driven by Th2 cells, and in the chronic phase, it is transformed into Th1, Th17 and Th22 cells (79). After homing to the inflammatory areas of the skin, TEM cells release cytokines including IL-4, IL-13, IL-31, etc. These cytokines, especially Th2 cytokines, are involved in the inflammatory response and itching in AD recurrence by activating the JAK/STAT pathway (80). The binding of IL-4 and IL-4R activates and phosphorylates JAK1 and JAK3, thereby activating STAT6 (81). As a key factor in the onset of AD, IL-4 and IL13 can promote Th2 cells to release inflammatory factors and recruit eosinophils and mast cells (82). Studies have shown that IL-4 is involved in the sensitization of itching by activating JAK1 (83). In addition, IL-4 and IL13 inhibit the production of antimicrobial peptides, thereby affecting skin barrier function and impairing the normal response of the skin to environmental pathogens (84). IL-31 is a pro-pruritus cytokine. After binding to receptors on eosinophils and keratinocytes (85), it signals through JAK1, JAK2, STAT1, STAT3 and STAT5 (86) to stimulate the secretion of pro-inflammatory cytokines and participate in the pathogenesis of atopic dermatitis. Therefore, treatment targeting the JAK-STAT pathway may reduce these signals and demonstrate therapeutic effects by blocking multiple immune pathways associated with AD. Local and systemic JAK inhibitors with different selectivity have emerged as potential therapeutic options for the treatment of AD (87).

## 6 Discussion

AD is a common chronic, inflammatory and recurrent skin disease characterized by infiltration of various immune cells, mainly lymphocytes, into the dermis. In this paper, by analyzing the research status of lymphocyte skin homing in AD, it is found that T cell homing to the skin plays a vital role in the recurrence of AD. The mediation of related molecules is indispensable in the process of skin-homing memory T cells migrating to the dermis. In the transendothelial migration of lymphocytes, CLA, integrin family LFA-1, VLA-4 and their ligands E-selectin, ICAM-1, and VCAM-1 on the surface of vascular endothelial cells are involved in mediating the adhesion and rolling process of skin-homing memory T cells (16). The interaction between chemokines CCL17 and CCL27 expressed on CLA+ T cells and their receptors CCR4 and CCR10 mediate T cells to stay and eventually cross vascular endothelial cells (17). Due to the critical role of related molecules in the recurrence of AD, directly or indirectly down-regulating the level of associated molecules in AD or inhibiting intermolecular binding to inhibit TEM homing is becoming a new method for the treatment of inflammatory skin diseases, namely AD. At the same time, the possible functional redundancy between molecules also suggests the necessity of blocking multiple pathways of treatment, providing a reference for the treatment of AD.

In addition to TEM, there are two other subtypes of memory T cells, Central Memory T cell (TCM) and Tissue Resident Memory T Cells (TRM). TCM mainly exists in blood circulation and stimulating lymphoid organ (88, 89), and is not involved in skin homing behavior in AD. In contrast, studies have shown that TRM also expresses skin-homing receptor CCR10 in AD (65, 90). In addition, TRM in AD patients can also secrete a variety of cytokines, including IL-4, IL-13, IL-17, IL-22, and IFN-γ, which play a key role in the persistent recurrent inflammatory response of AD (91–93). In AD patients, similar T cell receptor repertoires are found in both lesional and non-lesional skin, and this T cell receptor repertoire remains unchanged after four months of effective anti-inflammatory treatment. This indicates that TRM cells are present in both lesional and non-lesional skin (94). Recent studies have found that in the non-lesional skin of AD patients, compared with healthy skin, the expression of T cell-related mediators is increased, the expression of Th2/Th22/Th17-related genes is unbalanced, and the terminal differentiation process of keratinocytes is impaired (95–97). These changes indicate abnormal immune function and skin barrier dysfunction, respectively. Recent studies have found that local CD4+ TRM cells are vital in driving early inflammatory responses during AD recurrence. These cells may release specific cytokines and induce the expression of corresponding chemokines to coordinate the recruitment of neutrophils to the re-attack site on the skin (93). Despite these advances, the exact mechanism of TRM in AD remains to be further studied to provide new methods for the treatment of this intractable skin inflammatory disease.

In addition to T cells, other lymphocytes also have homing phenomena, which may be related to the mechanism of AD recurrence. NK cells also express CLA and other skin homing receptors (98). Recent studies have shown that the skin expression of NK cell markers NCAM-1/CD56 and Pan-granzyme is increased in AD, confirming that skin homing mainly occurs in severe AD (99). Granzyme may play a key role in the occurrence and persistence of AD inflammation by regulating the innate response (100). Despite these advances, the exact mechanism of action of these cells in AD remains to be further explored, with the hope of providing a new therapeutic approach for the treatment of this persistent skin inflammatory disease.
